# The Antler Cycle and Fecal Testosterone of Male Sambar Deer *Rusa unicolor unicolor* at the Horton Plains National Park in Sri Lanka

**DOI:** 10.1155/2020/6903407

**Published:** 2020-06-13

**Authors:** D. S. Weerasekera, S. J. Perera, D. K. K. Nanayakkara, H. M. S. S. Herath, A. N. L. Rathnasekara, K. B. Ranawana

**Affiliations:** ^1^Postgraduate Institute of Science, University of Peradeniya, Peradeniya, Sri Lanka; ^2^Department of Natural Resources, Faculty of Applied Sciences, Sabaragamuwa University, Belihuloya, Sri Lanka; ^3^Nuclear Medicine Unit, Faculty of Medicine, University of Peradeniya, Peradeniya, Sri Lanka; ^4^Epsilon Crest Research, 6/6.67 Ward Place, Colombo 07, Sri Lanka; ^5^Department of Zoology, Faculty of Science, University of Peradeniya, Peradeniya, Sri Lanka

## Abstract

This study is aimed at evaluating the relationship between endogenous testosterone levels and antler development in male sambar deer (*Rusa unicolor unicolor*) inhabiting the Horton Plains National Park, Sri Lanka. Seven antler growth stages of sambar were documented based on phenotypic observations for the first time in Sri Lanka as (a) cast, (b) growing 1—single spike, (c) growing 2—antler fork into a Y as the second tine appears, (d) growing 3—velvet begins to harden as the third tine appears, (e) growth completed—velvet shedding begins, (f) hard antler, and (g) casting. Fecal samples were collected every month for a period of eighteen months from male sambar deer in different stages of the antler growth cycle, feeding in the wet *patana* grasslands of the park, and the fecal testosterone level was estimated by radioimmunoassay. Ten animals were randomly selected from each antler stage for the experiment. The results disclose that the highest concentrations of testosterone were recorded in the hard antler stage. Velvet shedding was preceded by an increase in the testosterone level, and it is the sudden drop in the testosterone concentration which triggers the antler casting. The casting stage corresponded with the lowest mean testosterone concentration. Although the study was able to conclude a clear relationship between the fecal testosterone levels of the male sambar deer in the Horton Plains National Park and their antler stages, there is no clear seasonality for the antler cycle.

## 1. Introduction

Sambar (*Rusa unicolor unicolor*) is the most widely spread deer in the Asian continent [1]. Much of the knowledge on reproductive biology of the deer family (Mammalia: Artiodactyla: Cervidae) has been originated from studies of temperate species that exhibit a distinct seasonal cycle of reproductive behavior and antler growth in males [2].

Antlers are growing from the skull, only in male deer, with a solid calcified core and are unique in that they go through an annual cycle of rapid growth in preparation for the rutting season, after which they are casted [3]. Antlers are casted and regrown from a blastema annually into three-branched structures of cartilage and bone that are used for sparing and attraction in adult sambar deer [4]. Most of in vitro studies on the effects of testosterone in male deer on the bone growth and stages of their antler cycle have tested with cells sampled after casting stage of the antler, while some experiments have also been performed on the developing pedicle and primary antler tissue [5], all supporting the effect of testosterone on antler growth. The effects of testosterone in growing antler tissue might be mediated via local aromatization to estradiol as it generally occurs in many other bone tissues [5, 6]. However, other experiments show a relationship between the testicular volume and the growth of the antlers [7]. Further, the relationship between the antler growth has been examined with hormones secreted by testes in different antler stages [4]. In deer testicular atrophy, the epididymis was comparatively larger than testes [7]. Histologically, a substantial reduction, or absence, of germinal epithelium and absence of spermatozoa have been described in testicular atrophy [8]. When it comes to seasonal breeding in deer from temperate regions, the cycle of rutting and related behavior together with growth and casting of antlers is associated with secretion of testosterone by the testis [4, 9]. Changes in testosterone concentration, diameter of the testicles and mass of the body have been studied in relation to the antler cycles of such deer stags [9]. Thus, it is well established that reproductive steroid levels contribute to reproduction of animals [10].

Hormonal levels can be estimated in animals by using plasma or fecal matter [11]. Estimating the hormone levels using fecal steroid assays provides a noninvasive method, which have the capacity to develop long-term means of determining adrenocortical, testicular hormones in many vertebrate taxa [12]. However, studies on reproductive biology and the antler cycle conducted in tropical regions are scarce particularly on wild deer populations, while almost nothing is known from Sri Lanka. A single such study available on sambar deer in India has only been conducted on captive animals in the Thrissur Museum and Zoo, Kerala, India, for a period of four months in 2012 [13]. Nevertheless, the sambar population in the Horton Plains National Park (HPNP) is unique among other such populations in India and Sri Lanka by (a) aggregating into large herds counting to about 20 individuals in average opposed to general herd size of two to four and (b) by being predominantly grazers as opposed to the browsing behavior shown elsewhere [14]. Hence, the current field study attempted to study the relationship between antler stages and testosterone concentration in fecal matter, on the unique wild population of sambar deer in HPNP, Sri Lanka. This is the first time that fecal testosterone concentration was monitored by using radioimmunoassay (RIA) related to antler stages in wild sambar deer populations in the region.

Further, the current knowledge of the antler cycle of the wild sambar deer population in the HPNP is largely incomplete, while its seasonality has not been well established. Although a clear seasonality of sexual and antler cycles is known from cervids in temperate regions [15], our preliminary observations on this population having calves throughout the year suggest a nonseasonal breeding, while an increased emergence of new antlers was recorded after February, which is the month where many casting animals were recorded (Weerasekera, pers. obs.). Therefore, while exploring the relationship between antler stages and fecal testosterone concentration being the main aim, we also attempted to record any seasonality of the testosterone levels in sambar males. We hope this new knowledge could also provide important information for the park management in a presumably increasing population of sambar deer, with a limited availability of grasslands.

## 2. Materials and Methods

### 2.1. Study Area and the Antler Cycle

The study was carried out in Horton Plains National Park (HPNP), Sri Lanka, for a period of eighteen months (January 2018-June 2019). The HPNP was chosen for this study because of the large number of sambar deer inhabiting the park's wet *patana* grasslands. A period of about 30 man-days of *ad-libitum* preliminary observations [16] were conducted over a one-year period prior to the study, in order to understand the behavior of sambar deer in the HPNP and to record different stages of its antler cycle. There were over 1000 sambar deer in the HPNP, during the commencement of the study (Weerasekera and Perera, pers. obs.). Antler growth categories were identified by physical appearance of the antlers, especially the number of tines per antler and the length of the main beam [17], while the old males with irregular antler casting were not taken to the experiment. Additional characters that were considered for this study were the size and shape of the body, in order to estimate their age [18].

### 2.2. Fecal Sample Collection

During the eighteen-month sampling period, the HPNP was visited at monthly intervals where sambar deer at the age between three to five years [19], in different antler stages, were opportunistically sampled. Whenever a male sambar that could readily be classified into an identified antler stage and fulfilling the age criteria was observed to defecate, their fecal samples were collected as soon as possible. Ten such fecal samples belonging to each antler stage were collected, which took eighteen consecutive months to complete the expected number of samples. Spike bucks and two spiked bucks (males below three years of age) and old bucks with irregular antler casting (males above five years of age) were excluded from sampling. Age was identified by using standard features such as antler length and body condition [18]. Collected fecal pellets were labeled with the antler stage and the date of collection and kept in polyethylene covers and stored in an ice box during the transportation. In the laboratory, they were stored in -20°C until extraction for RIA analysis. We ensured that fecal samples were collected less than half an hour of voidance and kept in ice, in order not to let bacterial metabolism start affecting the concentration of fecal hormones.

### 2.3. Method Validation for Sambar Deer Fecal Testosterone

First, to assess the effectiveness of extracting testosterone metabolites from the sambar deer feces, we measured the recovery of radioactivity for each antler stage. In order to validate the samples, 10,000 cpm of ^125^I testosterone was added to 0.2 g of dry feces of sambar deer. After incubation at room temperature for 1 h, we continued with the extraction of testosterone from solid-phase by following methanol extraction [20]. A large mass of feces was thoroughly mixed, and we weighed 10 aliquots of 0.5 ml wet feces. Then, a testosterone tracer of 18,000 cpm ^125^I was added to each aliquot. After incubating aliquots at room temperature for 1 h, we positioned aliquots in 3 ml methanol/acetone solution (4 : 1) and extracted each aliquot using the same methods for each antler stage. We measured the recovery of radioactivity on elution with a gamma meter.

As the second step, the ^125^I testosterone RIA antibody kit was validated for sambar deer. To test for parallelism with the regular curve, we ran serial dilutions of sambar deer fecal extract pools by spiking each norm with an aliquot from the respective fecal pool and running them as samples and calculated the mean assay accuracy for each sample. For the assay, we calculated variance coefficients for all the samples intra- and inter-assay.

In the third step, we compared testosterone concentrations between male calves and three spiked bucks as a physiological validation to our methods. While we did not expect age-related changes to be the same, we expected a clear variation in which male calves will show lower testosterone metabolite concentrations than the adult bucks [21].

### 2.4. Fecal Steroid Extraction and Radioimmunoassay

Frozen fecal specimens were dried in a standard oven, and each sample was carefully powdered and blended. A subsample weighing 0.2 g was mixed in a test tube with 5 ml of 90% ethanol and briefly vortexed. Tubes were then boiled in a water bath (90°C) for 20 minutes, adding ethanol to prevent dry cooking [22].

The extract was preboiled with 90% ethanol and centrifuged for 20 minutes at 1500 rpm. The extraction was then poured into another storage vial, and a further 5 ml of 90% ethanol was added to the remaining fecal powder and vortexed for 30 seconds and centrifuged for 15 minutes at 1500 rpm. Combined and dried down, the first and second extracts were reconstituted in one ml of methanol and vortexed for a short time. The methanol samples were placed at -20°C until the RIA assessment using the ^125^I testosterone RIA kit [13], and the concentration of testosterone was associated with the stages of the antler cycle. The lower limit of detection in this assay was 0.2 ng g^−1^ fecal powder, and the inter- and intra-assay coefficient of variation was <12.4%.

## 3. Results and Discussion

Sambar deer herds in the HPNP were observed throughout the study period, and those stags belonging to different antler stages were opportunistically included in the study. The present study provided the first seasonal evaluation to monitor testicular steroidogenic activity in sambar deer in the HPNP, employing a noninvasive technique. Testosterone concentrations were measured in fecal samples using a simple extraction method and a commercial RIA kit [10]. Initially, we validated a practical and appropriate procedure for monitoring fecal testosterone in sambar deer stags, followed by the establishment of a relationship between antler stages and fecal testosterone concentration for the unique wild population of sambar deer in the HPNP, further attempting to record any seasonality of the testosterone levels in sambar males.

### 3.1. The Antler Cycle of Sambar Deer

**Figure 1:**
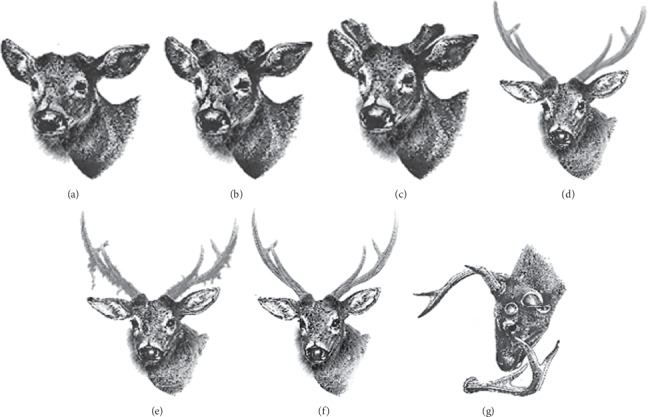
Antler stages of sambar deer (*Rusa unicolor unicolor*) recorded from Horton Plains National Park, Sri Lanka: (a) cast, (b) growing 1—single spike, (c) growing 2—antler fork into a Y as the second tine appears, (d) growing 3—velvet begins to harden as the third tine appears, (e) growth completed—velvet shedding begins, (f) hard antler, and (g) casting.

Initially, we identified distinct stages of the antler growth from our preliminary *ad libitum* observations on sambar males in the HPNP. Hence, the antler cycle of sambar deer is recorded here for the first time in Sri Lanka with seven antler growth stages, namely, (a) cast, (b) growing 1—single spike, (c) growing 2—antler fork into a Y as the second tine appears, (d) growing 3—velvet begins to harden as the third tine appears, (e) growth completed—velvet shedding begins, (f) hard antler, and (g) casting (Figure 1).

Any of the seven stages of the antler cycle could be observed at any given point of time during the year, indicating no marked seasonality in antler cycle among the sambar deer in the HPNP [5, 14]. The argument by Shipka et al. [23] that “sexual and antler cycles of deer species near the equator, where day length differs only slightly, are asynchronous” is supportive of our findings on sambar deer stags in the HPNP. This observation is also supported by Sankar and Acharya [24] who recorded spotted deer (*Axis axis*) in southern Indian region is not seasonal in their antler cycle. The seasonality in the antler cycle in herds of red deer in the temperate region is well established by Wilson et al. [25], Loudon et al. [26], and Curlewis et al. [15]. Although a single observation by Heckeberg [27] has recorded cervids in equatorial region also to be seasonal in their antler cycle, our results supported by Shipka et al. [23] and Sankar and Acharya [24] strongly suggests this seasonality to be diffused in the tropics, recommending a reinvestigation on Heckeberg's observation.

### 3.2. Antler Stages and Fecal Testosterone of Male Sambar Deer

**Figure 2:**
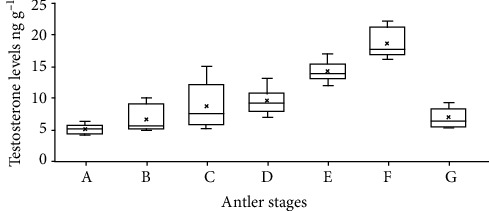
Box and whisker plots for mean fecal testosterone levels in ng g^−1^ during different antler stages of sambar deer (*Rusa unicolor unicolor*) monitored from January 2018 to June 2019 in the Horton Plains National Park. Testosterone was obtained and measured from radioimmunoassay using methanol. Fecal matter analyzed belongs to either of the antler stages: (a) cast, (b) growing 1—single spike, (c) growing 2—antler fork into a Y as the second tine appears, (d) growing 3—velvet begins to harden as the third tine appears, (e) growth completed—velvet shedding begins, (f) hard antler, and (g) casting.

Concentration of fecal testosterone is clearly associated with the stages of antler development in cervids [28]. Here, we present a clear relationship between the mean fecal testosterone metabolite levels in dry feces of male sambar deer in the HPNP with the stages of their antler cycle (Table 1; Figure 2).

As tabulated in Table 1, the maximum mean fecal testosterone level (18.56 ± 2.17 ng g^−1^) was recorded in the hard antler stage of sambar males in the HPNP (Figure 2(f)), corresponding to the courtship behavioral events, mounting, and copulation, while the minimum mean fecal testosterone level (4.58 ± 0.71 ng g^−1^) was in the cast antler stage (Figure 2(a)). The mean fecal testosterone level of male sambar deer in growing 1 (single spike) antler stage was 6.58 ± 01.97 ng g^−1^ (Figure 2(b)). Sambar stags in growing 2 (antler fork into a Y as the second tine appears) antler stage show an elevation in the mean fecal testosterone level to 8.68 ± 03.73 ng g^−1^ (Figure 2(c)), while it continued to rise towards sambar stags in growing 3 (velvet begins to harden as the third tine appears) antler stage (9.57 ± 1.84 ng g^−1^; Figure 2(d)). Mean testosterone concentration in dry feces of male sambar deer in growth completed (velvet shedding begins) stage was markedly high as 14.18 ± 1.76 ng g^−1^ (Figure 2(e)), and the stags in hard antler stage maintained the highest mean testosterone level as stated above (Figure 2(f)). This was a well-marked increase reaching the maximum of mean testosterone level in dried fecal matter of the stags in hard antler stage, in comparison to all other stages, whereas the mean fecal testosterone concentration of male sambar deer in casting stage dropped rapidly (Figure 2(g)).

Our preliminary observations on sambar deer in the HPNP revealed that their antlers were in velvet growth for a quite long duration of seven to eight months, mostly around April to September, while velvet antlers are not rare to be seen in other months as the antler cycle of the population is not completely synchronized. The hard antlers were recorded throughout the breeding season which lasted for about four to five months, predominantly from September to January. This was followed by the antler casting stage, where the deer cast their antlers either in a fight or hitting against a tree or any other hard structure [29]. The results reported here are similar to previous findings as the fallow deer (*Dama dama*) stags are recorded with peak fecal testosterone concentrations during November–January [9], while November to January has been identified as the breading season of sambar deer elsewhere in the tropics [2, 6].

Regarding the annual activation of the reproductive system, it has been speculated that this model of testosterone secretion is a relic of the original reproductive pattern of earlier cervids, which developed in the semitropical regions [12]. This hypothesis could explain why males of some cervid species living in tropical and subtropical areas, including pampas deer (*Ozotoceros bezoarticus*), have well-defined and synchronous antler cycle, even though sexual activity may happen throughout the year. Another important aspect associated with testosterone secretion in cervid species is the display of sexual behavior. Bubenik et al. [30] stated that in white-tailed deer (*Odocoileus virginianus*), maximal testosterone concentrations were not necessary for the process of spermatogenesis but were essential for manifestation of reproductive behavior. Conversely, sexual and aggressive behaviors in Eld's deer (*Rucervus eldii*) bucks were the highest when testosterone concentrations and testis size started declining [31].

Kierdorf and Kierdorf [32] noted that antlers are generally casted after the rutting/mating season, while Price et al. [33] noted that elk deer (*Cervus canadensis*) shed antlers during sparing show, prior to the mating season in each spring, within the temperate zone with seasonal climates. However, in sambar deer of the HPNP, it was observed that most of the stags lost both their antlers on the same day, while some individuals were observed to cast their two antlers with a gap of 2-3 days [33]. Casting stage, which correspond to low concentration of fecal testosterone did not last long for a given individual, which will be over within a maximum of three days. As the antler growth is a rapid continuous cycle, the beginning of the next pair of antlers of the same individual set in without a long interval.

In white-tailed deer (*Odocoileus virginianus*), the testosterone exhibits the highest concentration during the mating season [34], while in red deer (*Cervus elaphus*), the highest testosterone concentration was recorded mainly during the rutting, at the beginning of the mating season [35]. Findings on red deer stags revealed that mean serum testosterone concentrations increased during the breeding season up to about one to two weeks prior to the peak of the rut [36]. However, Ditchkoff et al. [37] discovered that even in white-tailed deer (*Odocoileus virginianus*), testosterone showed the highest concentration during the rut. Uvíra et al. [38] and Stewart et al. [31] discovered that high concentrations of testosterone are observed at the beginning of a series of biological activities in many mammals followed by a continuous decrease after the activity period. Antler development cycles are strongly linked to staggered sexual cycles and can be ascribed directly to differences in seasonal photoperiod affecting gonadal steroidogenic activity in temperate regions [39]. Testosterone levels peak immediately before rut, and it is the rapid decline in its level that causes antler casting. Antler growth occurs at a low testosterone concentration and is seen increasing when the antler growth nears completion. Velvet shedding and antler hardening are the consequences of high testosterone levels [39].

## 4. Conclusion

The fecal testosterone levels of the seven antler stages of male sambar deer in the HPNP tested by RIA indicate a strong relationship of testosterone with different stages of the antler cycle. The results obtained in this study were in agreement with similar research work carried out in other species of deer which were primarily with temperate origins, despite lacking a pronounced seasonality. The sambar in the hard antler stage showed a higher mean concentration of testosterone in relation to the antler cycle's velvet stage, while such differences in testosterone levels are in agreement with the results of many other investigators. Such studies also agree that the antler casting is caused by the immediate drop in testosterone concentration. The shedding of velvet was also accompanied by an increase in the level of testosterone which emphasized its function. Although the study was able to conclude a clear relationship with testosterone concentration and stages of the antler cycle of sambar deer (*Rusa unicolor unicolor*) in the HPNP with tropical montane climate, there is no clear seasonality for the antler cycle as reported in many temperate ancestral cervids.

## Figures and Tables

**Table 1:** Distribution of fecal testosterone concentration (ng g^−1^) at each antler stage of sambar deer in Horton Plains National Park, Sri Lanka.

Stage of the antler cycle	Fecal testosterone concentration (ng g^−1^)			
	Mean ± SE	Minimum	Maximum	Range
Cast	4.58 ± 0.71	4	5.6	1.6
Growing 1—single spike	6.58 ± 1.49	5	10	5
Growing 2—antler fork into a Y as the second tine appears	8.68 ± 3.73	5.2	15	9.8
Growing 3—velvet begins to harden as the third tine appears	9.57 ± 1.84	7	13	6
Growth completed—velvet shedding begins	14.18 ± 1.76	12	17	5
Hard antler	18.56 ± 2.17	16	22	6
Casting	6.84 ± 1.58	5.1	9.2	4.1

## Data Availability

The underlying raw data used to support the findings of this study are available from the corresponding author upon request.
